# Targeting Signaling Pathway Downstream of RIG-I/MAVS in the CNS Stimulates Production of Endogenous Type I IFN and Suppresses EAE

**DOI:** 10.3390/ijms231911292

**Published:** 2022-09-25

**Authors:** Anne K. Kronborg Hansen, Magdalena Dubik, Joanna Marczynska, Bhavya Ojha, Estanislao Nistal-Villán, Gloria González Aseguinolaza, Dina S. Arengoth, Trevor Owens, Reza Khorooshi

**Affiliations:** 1 Department of Neurobiology Research, Institute of Molecular Medicine, University of Southern Denmark, 5000 Odense, Denmark; 2 Microbiology Section, Department of Pharmaceutical and Health Science, Faculty of Pharmacy, University CEU San Pablo, Campus Montepríncipe, 28003 Madrid, Spain; 3 Gene Therapy and Regulation of Gene Expression Program, Center for Applied Medical Research (CIMA), University of Navarra, 31008 Pamplona, Spain; 4 IdiSNA Navarra Institute for Health Research, 31008 Pamplona, Spain

**Keywords:** type I interferon, RIG-I, experimental autoimmune encephalomyelitis, RIG-I, MAVS, 2CARD-MAVS200

## Abstract

Type I interferons (IFN), including IFNβ, play a protective role in multiple sclerosis (MS) and its animal model, experimental autoimmune encephalomyelitis (EAE). Type I IFNs are induced by the stimulation of innate signaling, including via cytoplasmic RIG-I-like receptors. In the present study, we investigated the potential effect of a chimeric protein containing the key domain of RIG-I signaling in the production of CNS endogenous IFNβ and asked whether this would exert a therapeutic effect against EAE. We intrathecally administered an adeno-associated virus vector (AAV) encoding a fusion protein comprising RIG-I 2CARD domains (C) and the first 200 amino acids of mitochondrial antiviral-signaling protein (MAVS) (M) (AAV-CM). In vivo imaging in IFNβ/luciferase reporter mice revealed that a single intrathecal injection of AAV-CM resulted in dose-dependent and sustained IFNβ expression within the CNS. IFNβ expression was significantly increased for 7 days. Immunofluorescent staining in IFNβ-YFP reporter mice revealed extraparenchymal CD45+ cells, choroid plexus, and astrocytes as sources of IFNβ. Moreover, intrathecal administration of AAV-CM at the onset of EAE induced the suppression of EAE, which was IFN-I-dependent. These findings suggest that accessing the signaling pathway downstream of RIG-I represents a promising therapeutic strategy for inflammatory CNS diseases, such as MS.

## 1. Introduction

Interferon beta (IFNβ), a member of the type I IFN family, has been shown to play a protective role in multiple sclerosis (MS) and experimental autoimmune encephalomyelitis (EAE), the most common animal model used to understand aspects of MS [1]. Type I IFNs are induced by the activation of innate receptors, including Toll-like receptors (TLR) and retinoic acid-inducible gene I (RIG-I), that recognize pathogen- or danger-specific signatures [2]. Innate receptors constitute one of the mechanisms involved in the regulation of inflammation in the CNS, and accessing these pathways may provide a potential therapeutic target for regulating autoimmune inflammation in MS.

Stimulation of innate receptors within the CNS has been shown to induce IFNβ and infiltration of myeloid cells with an EAE-suppressive function [3]. We previously showed that a single intrathecal injection of different innate ligands induced transient expression of endogenous IFNβ in the CNS, recruited myeloid cells to the CNS and transiently suppressed EAE [4,5].

Signaling via RIG-I, a cytoplasmic RNA sensor associated with mitochondrial antiviral-signaling protein (MAVS), plays a critical role in the induction of IFNβ [6,7] and has protective functions in EAE [8]. In the present study, we targeted the signaling pathway downstream of RIG-I in order to stimulate the production of endogenous IFNβ.

We intrathecally administered an adeno-associated virus (AAV) vector encoding a fusion protein (CM) comprising RIG-I 2CARD domains and the first 200 amino acids of MAVS (AAV-CM) [9]. CM acts downstream of RIG-I and MAVS to induce a plethora of immunoregulatory mediators, including IFNβ [9]. We used this AAV approach to ask whether overexpression of CM in the CNS induces IFNβ and how this would exert a therapeutic effect against EAE.

Our findings show that intrathecal treatment with CM induced IFNβ in extraparenchymal blood-derived cells, choroid plexus and astrocytes, and suppressed EAE in an Interferon-α/β receptor 1 (IFNAR1)-dependent manner. We link the protective action of CM to its ability to induce CNS-endogenous IFNβ via stimulation of previously unexplored RIG-I/MAVS intracellular signaling pathway in the CNS. Our findings suggest that targeting such signaling pathways can be exploited for the development of novel therapeutic approaches for inflammatory CNS diseases, such as MS.

## 2. Results

### 2.1. Intrathecal AAV-CM Induced IFN Beta Response in the CNS

**Figure 1:**
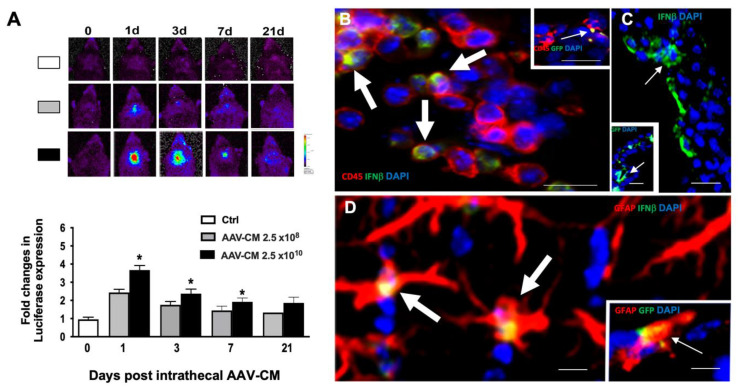
Intrathecal AAV-CM induces sustained IFNβ. (**A**) IFNβ expression in the brain was evaluated in luciferase reporter mice by in vivo imaging and showed that intrathecal administration of AAV-CM induced IFNβ in the CNS of mice was dose-dependent. In vivo imaging of IFNβ/luciferase reporter mice that received intrathecal AAV-CM. The level of IFNβ was significantly increased at 1-, 3- and 7-days post injection and was detectable up to 21 days post injection. *n* = 3–4 in each group. B–D) Micrographs of brain sections from mice that received AAV-CM or AAV-GFP intrathecally. Nuclei were stained with DAPI (blue). (**B**) Co-localization (arrows) of IFNYβ/YFP + (green) and extraparenchymal CD45 + cells (red) in mice are shown. Insert shows the co-localization of GFP+ (green) and extraparenchymal CD45 + cells. (**C**) IFNYβ/YFP + (green, arrow) or GFP+ cells (green in insert, arrow) in the choroid plexus. (**D**) Co-localization (arrows) of IFNYβ/YFP+ (green) or GFP+ cells (green in insert) and GFAP+ (astrocytes) in the perivascular area of the lateral ventricle. Data are presented as mean ± SEM. The results were analyzed using the two-tailed Mann–Whitney u-test. * *p* < 0.05, Scale bars. 10 µm.

We examined whether intrathecal administration of AAV-CM induces IFNβ in the CNS of mice that express a luciferase gene under control of the IFNβ promoter [10]. AAV-CM and AAV-GFP were injected intrathecally, and luciferase activity was measured at 1-, 3-, 7- and 21-days post administration. In vivo imaging revealed that a single intrathecal injection of AAV-CM resulted in a significant increase of IFNβ expression within the CNS at 1-, 3- and 7-days post injection (Figure 1A). Furthermore, IFNβ expression remained detectable for an additional 14 days post intrathecal injection (Figure 1A). AAV-CM-induced IFNβ response was dose-dependent (Figure 1A). As expected, intrathecal AAV-GFP did not induce IFNβ at any time point.

In order to investigate the localization and cellular sources of IFN-β in response to AAV-CM, IFNβ/yellow fluorescent protein (YFP) knock-in mice were used [11]. C57BL/6 mice were used for the detection of GFP in AAV-GFP-treated mice. Brains were examined for IFNβ or GFP expression at 1-, 3-, and 7-days post administration [5]. Double immunostaining showed IFNβ colocalization with CD45+ cells that were distributed in the leptomeningeal space at 1 day post AAV-CM treatment (Figure 1B, Arrows). CD45-expressing cells were more abundant in mice that had received AAV-CM compared to AAV-GFP-treated mice at 1 day post injection. At this time point, we observed a few leptomeningeal CD45+ cells that colocalized with GFP in intrathecally AAV-GFP-treated mice, indicating that CD45+ cells expressed the vector (Figure 1 insert in B, Arrow).

At both 3- and 7- days post intrathecal AAV-CM treatment, we observed only a few CD45+ cells in the leptomeningeal space. IFNβ+ cells at 3- and 7-days post AAV-CM- treatment were mainly found in the choroid plexus and in the perivascular area where they co-colocalized with GFAP+ astrocytes (Figure 1C,D). At these time points, GFP+ cells were also observed in the choroid plexus and in the perivascular area, where they were also co-colocalized with GFAP+ astrocytes (insert in Figure 1C,D, Arrows). Together, these findings show that AAV-CM infected leptomeningeal CD45 + cells, choroid plexus and astrocytes, and induced their expression of IFNβ.

### 2.2. Intrathecal AAV-CM Treatment Enhanced CNS Recruitment of Myeloid Cells

**Figure 2:**
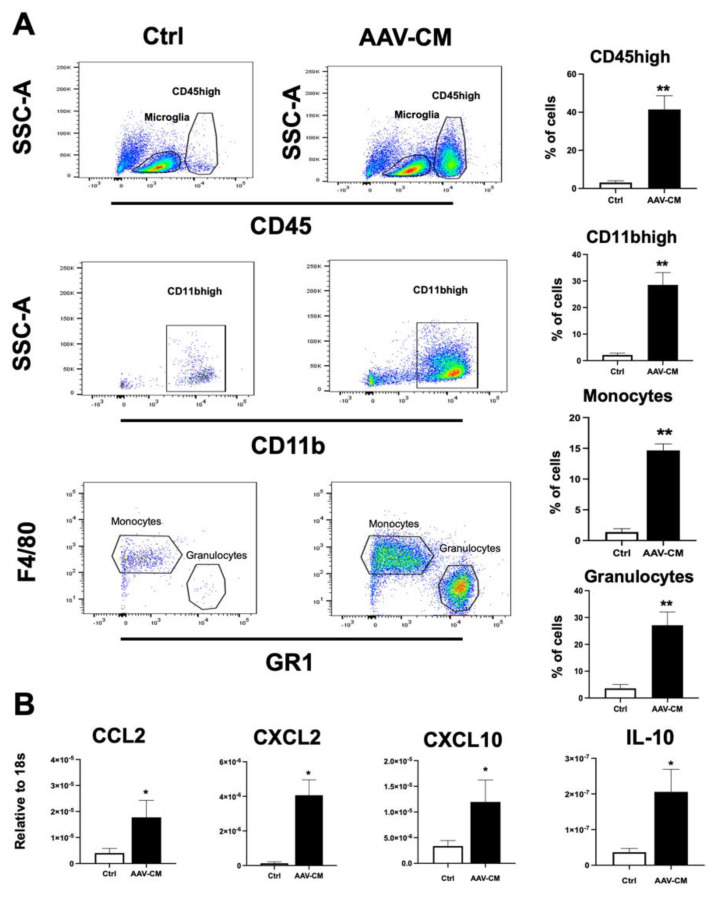
Intrathecal AAV-CM recruits myeloid cells to the healthy CNS. (**A**) Flow cytometric gating strategy to distinguish CD45^high^ leukocytes from CD45^dim^ microglia, and CD11b^high^, macrophages/monocytes (CD45^hi^CD11b^hi^GR1^low/-^F4/80^+^), and granulocytes (CD45^hi^CD11b^hi^GR1^hi^F4/80^-^). The proportion of CD45^high^, CD11b^high^, monocytes and granulocytes were significantly increased in the CNS tissues of mice upon intrathecal AAV-CM treatment (*n* = 4–6 per group). (**B**) RT-qPCR analysis of brains showed CCL2, CXCL2, CXCL10 and IL-10 significantly induced upon intrathecal AAV-CM treatment at 1 day post dose (*n* = 4–6). control (ctrl). Data are presented as mean ± SEM. The results were analyzed using the two-tailed Mann–Whitney u-test. * *p* < 0.05, ** *p* < 0.01.

The results from immunostaining suggested that AAV-CM induces CNS recruitment of CD45+ cells, including polymorphonuclear cells, at 1 day post treatment. To examine this further, mice were administered AAV-CM or a control vector by intrathecal injection via cisterna magna, and CNS tissues were analyzed by flow cytometry at 1 day post injection. Blood-derived myeloid cells were distinguished from microglia by CD45^high^ versus CD45^dim^ discrimination (Figure 2A). We found that AAV-CM induced a significant increase in the proportion of CD45^high^ cells in CNS tissues (Figure 2A), similar to that observed by immunostaining (Figure 1B). Moreover, AAV-CM induced significant recruitment of myeloid cells (CD45^hi^CD11b^hi^), including monocytes (CD45^hi^CD11b^hi^GR1^low/-^F4/80^+^) and granulocytes (CD45^hi^CD11b^hi^GR1^hi^F4/80^−^) (Figure 2A). Initial studies showed that AAV-CM-induced infiltration of CD45^high^ cells response was dose-dependent (supplementary Figure S1). As expected, low numbers of CD45^high^ cells were detected in the AAV-GFP-treated control (Ctrl) mice.

These findings show that AAV-CM induces the recruitment of myeloid cells into the CNS. Therefore, we isolated RNA from CNS tissue for the RT-qPCR analysis of chemokines that recruit myeloid cells. Intrathecally, AAV-CM-treated mice showed significant upregulation in mRNA levels of CCL2, CXCL10 and CXCL2, involved in monocyte and neutrophil recruitment, at 1 day post dose (Figure 2B). The anti-inflammatory cytokine IL-10, which is induced by IFNAR1- and RIG-I signaling, was also upregulated in response to AAV-CM (Figure 2B).

### 2.3. Intrathecal AAV-CM Suppressed EAE in an IFNAR-Dependent Manner

**Figure 3:**
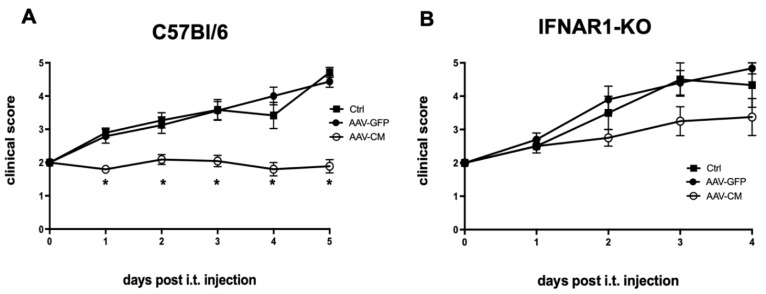
Intrathecal AAV-CM suppressed EAE in an IFNAR-dependent manner. C57BL/6 or IFNAR1-deficient mice were immunized with MOGp35–55 to induce EAE and at disease onset, they received intrathecal AAV-CM, AAV-GFP or PBS, and clinical signs were scored daily. (**A**) Mice that received AAV-CM showed marked attenuation of the disease compared to the control mice. Sick mice treated with intrathecal AAV-GFP showed similar symptoms to those in PBS-treated mice (*n* = 12–22 in each group). The data were pooled from three independent studies. (**B**) The protective effect of intrathecal AAV-CM was abrogated in IFNAR1-KO mice. (*n* = 4–5 per group). Data are presented as mean ± SEM. The results were analyzed using the two-tailed Mann–Whitney u-test. * *p* < 0.05.

We asked whether induction and prolonged production of endogenous IFNβ would exert a therapeutic effect against EAE. To investigate this, C57BL/6 mice were immunized with MOG p35–55 and randomized on the day of disease onset, which in all cases was the loss of tail tonus (grade 2). Mice were administered AAV-CM, AAV-GFP or PBS into the cisterna magna and evaluated for clinical symptoms over the following ten days. The mean clinical score showed a significant increase in AAV-GFP or PBS-treated mice from 1 day to 5 days but did not change in mice that received intrathecal injection of AAV-CM (Figure 3A). Importantly, the disease modulatory effect of intrathecal AAV-CM was abrogated in IFNAR1-deficient mice, in which disease symptoms in AAV-CM-treated mice worsened similarly to those in AAV-GFP- or PBS-treated mice (Figure 3B). For ethical reasons, mice were sacrificed when they reached grade 5 or if hind limb paralysis persisted for 2 days.

### 2.4. Intrathecal AAV-CM Altered Inflammatory Programs in the CNS of Mice with EAE

**Figure 4:**
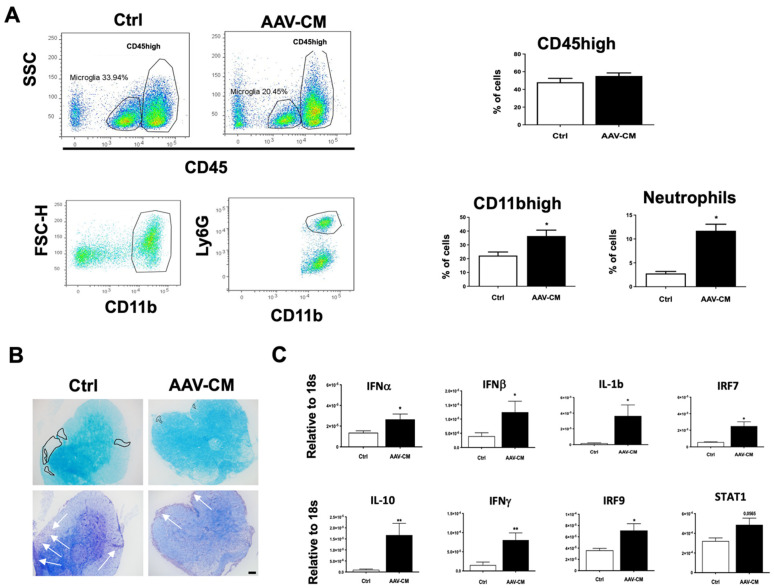
Intrathecal AAV-CM in mice with EAE induced the recruitment of myeloid cells, including neutrophils, to the CNS and reduced demyelination. (**A**) Flow cytometry profiles of brains of mice with EAE-treated with intrathecal AAV-CM or a control (ctrl). CD45high leukocyte populations are distinguished from CD45dim microglia. The proportion of CD45+ cells in the CNS remained similar in response to intrathecal AAV-CM treatment compared to the control EAE mice. Flow cytometric analysis revealed that the proportion of CD11bhigh and neutrophils were significantly increased upon intrathecal AAV-CM. *n* = 3–6 per group. (**B**) Images of lumbar spinal cord sections of mice with EAE stained with LFB and Cresyl violet. In the control group of mice, Cresyl violet staining showed cell infiltration into the parenchyma of the spinal cord (arrows), which was correlated with extensive loss of LFB (marked area) in corresponding areas. Mice with EAE that were treated with intrathecal AAV-CM showed cell accumulation in the meninges and reduced loss of LFB staining. Scale bar. 100 µm. (**C**) RT-qPCR analysis of brains showed IFNα, IFNβ, IFNγ, IL-10, IL-1β, IRF7 and IRF9 were significantly (STAT1, *p* < 0.0565) induced upon intrathecal AAV-CM treatment at 1 day post dose (*n* = 6–8). Data are presented as mean ± SEM. The results were analyzed using the two-tailed Mann–Whitney u-test. * *p* < 0.05, ** *p* < 0.01.

We asked whether and how intrathecal AAV-CM would impact the infiltration of immune cells into the CNS using flow cytometry analysis in mice with EAE. The results showed that the percentages of CD45^high^ cells were not different between the control and AAV-CM-treated mice (Figure 4A). However, further analysis of CD45^high^CD11b+ cells revealed that the proportions of infiltrating myeloid cell populations, including neutrophils, were significantly increased in CM-treated mice (Figure 4A), whereas the proportions of other populations that were examined remained unchanged (not shown).

The spinal cords of C57BL/6 mice with EAE were examined for demyelination and infiltration (Figure 4B). LFB staining revealed a loss of myelin in the parenchyma of the spinal cord in the control EAE mice. Cresyl violet staining showed infiltrating cells in the corresponding parenchymal areas in the control mice. Myelin loss was reduced by AAV-CM treatment, and infiltration in spinal cord sections was predominantly extraparenchymal in the meninges (Figure 4B).

To assess how the activation of RIG-I and IFNAR downstream signaling influenced CNS inflammatory programs in mice with EAE, we examined the expression of inflammation-associated mediators in response to AAV-CM using RT-qPCR. We found that levels of IFNα-, IFNβ-, IL10-, IL-1β and IFNγ mRNA, as well as IFNAR associated downstream signaling IRF7, IRF9 were significantly elevated in CNS tissue from AAV-CM-treated mice at 1 day post dose (Figure 4C).

## 3. Discussion

In this study, we have shown that targeting the signaling pathway downstream of RIG-I and MAVS stimulated the production of endogenous IFNβ in the CNS and exerted a therapeutic effect against EAE in an IFNAR1-dependent manner. Histopathology of control mice with EAE showed infiltrating cells in the spinal cord white matter, as well as myelin loss in corresponding areas. In contrast, infiltrating cells were predominantly found in the meninges of the spinal cord and myelin loss was minimal upon AAV-CM treatment.

Immunohistological and flow cytometry analysis showed the recruitment of CD45+ cells to the CNS in healthy mice upon AAV-CM administration. CD45 + cells included monocytes and granulocytes. Activation of innate receptors, including RIG-I signaling, has been shown to induce CCL2 and CXCL2, monocyte- and neutrophil- chemoattractants [12]. Here, we have demonstrated that both chemokines were upregulated in response to AAV-CM, supporting the idea that CM activated downstream RIG-I and MAVS signaling.

We have previously shown that the recruitment of immune cells into the CNS in response to innate receptor activation can protect against EAE, and that infiltrating CD45+ cells are a source of IFNβ [4,5]. In contrast to our previous work, where we observed a transient therapeutic effect on EAE by a single intrathecal injection of innate ligands [4,5,13], in the present study, there was a prolonged IFNβ expression as well as sustained protection against EAE.

Luciferase activity, in AAV-CM-treated IFNβ/luciferase reporter mice, was significantly increased during the first 7 days and was detectable for a further 14 days. A study by Aschauer et al. (2013) demonstrated that AAV8 effectively transduces cells of the CNS, particularly astrocytes [14]. Similarly, Pignataro and colleagues showed high AAV8 transduction efficiency within CNS tissue, including astrocytes and oligodendrocytes [15]. Our experiments showed co-localization of GFP with CD45 cells, cells of the choroid plexus, and astrocytes. As AAV8-CM and AAV8-GFP should transduce the same cells, our findings suggest that AAV-CM infected leptomeningeal CD45+ cells, choroid plexus and astrocytes, and induced their expression of IFNβ.

The RT-qPCR analysis showed increased levels of IFNβ and IL-10 in the CNS tissues of mice with EAE. Both IL-10 and IFNβ are known to play critical roles in the regulation of EAE and have been shown to contribute to the anti-inflammatory environment in the CNS [5,16]. IFNβ promotes immunosuppressive activity of myeloid cells [17] and it has been shown that type I IFN can drive the expression of IFNγ, which was also upregulated in our study [18,19]. Although normally considered pro-inflammatory, immunomodulatory roles for IFNγ have been proposed [20]. In our study, we found the levels of IFNα/β and IL10 to be increased more than IFNγ, suggesting that AAV-CM treatment shifted the inflammatory response toward a protective response.

Moreover, dysregulation of IL-10 has been associated with an enhanced risk for the development of autoimmune diseases [21]. Our previous study showed that neutrophils are one source of IL-10 [4]. Accumulating evidence suggests that neutrophils can acquire a suppressive phenotype under certain conditions and contribute to the regulation of inflammation [22]. We have previously shown that suppressive neutrophils can transfer protection against EAE [4]. In the present work, neutrophils were observed to be significantly increased in mice with EAE when treated with intrathecal AAV-CM; however, we did not examine whether the neutrophils in the present study contributed to the observed disease amelioration.

RIG-I recognizes single-stranded RNA. The downstream signaling of RIG-I involves MAVS and the activation of NF-κB, which regulates the expression of cytokines and chemokines including IFNβ and CXCL10 [23,24]. Accordingly, in our study, the level of CXCL10 increased in response to CM, suggesting the involvement of the NF-κB pathway in CM-induced CXCL10 expression. It has been shown that NF-kB-deficient mice are resistant to EAE, and activation of the NF-κB pathway exacerbates EAE [25,26]. Consistent with this, neutralizing IFN-inducible CXCL10 has been shown to exacerbate EAE [27] and increased EAE susceptibility has been observed in CXCL10-deficient mice [28].

The RT-qPCR analysis showed increased levels of message for IFNα, IRF7 and IRF9 in the CNS tissues of mice with EAE, indicating activated type I IFN-IFNAR signaling [29] Supporting this, the therapeutic effect of CM-induced type I IFN was absent in mice that lack IFNAR1 signaling.

## 4. Materials and Methods

### 4.1. Mice

Female albino (C57BL/6-Tyr^c−2J^) IFNβ^+/Δβ-luc^ mice (IFNβ/luciferase reporter mice) [10], Yellow Fluorescent Protein (YFP) (IFN-β^mob/mob^) IFNβ knock-in mice and IFNAR1-KO mice (C57BL/6 background) were all bred and housed in the Biomedical Laboratory, University of Denmark. Female C57BL/6j mice were purchased from Taconic Europe A/S (Lille Skensved, Denmark). All experiments were approved by the Danish Animal Experiments Inspectorate (approval number 2020–15-0201–00652).

### 4.2. EAE Induction

C57BL/6 and IFNAR1-KO mice were immunized as described previously [30] with 100 µL emulsion containing 100 µg myelin oligodendrocyte glycoprotein (MOG)p35–55 (TAG Copenhagen A/S, Denmark) in complete Freund’s adjuvant (BD Biosciences, Sparks, NV, USA) with 200 µg heat-inactivated *Mycobacterium tuberculosis* (BD Biosciences) injected subcutaneously into each hind flank. Mice received an intraperitoneal injection of *Bordetella pertussis* toxin (0.3 μg, Sigma-Aldrich, Brøndby, Denmark) at the time of immunization and 1-day post immunization. Mice were monitored daily for loss of body weight and EAE symptoms. The EAE grades were defined as follows: grade 0, no signs of disease; grade 1, weak or hooked tail; grade 2, floppy tail indicating complete loss of tonus; grade 3, floppy tail and hind limb paresis, grade 4: floppy tail and unilateral hind limb paralysis; grade 5, floppy tail and bilateral hind limb paralysis.

### 4.3. Intrathecal Injection

Mice were anesthetized by inhalation of isoflurane (Abbott Laboratories), and a 30-gauge needle (bent 55° with a 2 mm tip) attached to a 50 μL Hamilton syringe was used to perform an intrathecal injection of ssAAV8-EF-CARD-MAVS (AAV-CM) or ssAAV8-EF-Stuffer-eGFP-WPRE (AAV-GFP) [9] or phosphate-buffered saline (PBS). To determine an optimal dose for induction of IFNβ, mice received two different doses of AAV-CM. AAV-CM at a dose of 2.5 × 10^10^ or 2.5 × 10^8^ viral particles (vp)/animal, in a total volume of 10 µL, into the intrathecal space of the cisterna magna into the cerebrospinal fluid. The optimal dose for AAV-CM was determined based on the strongest induction of IFNβ expression in luciferase reporter mice by in vivo imaging. The results showed that the optimal dose for AAV-CM is 2.5 × 10^10^ vp/mouse and was used throughout the study. The dose of AAV-GFP was accordingly chosen at 2.5 × 10^10^ vp/mouse to match the AAV-CM dose.

### 4.4. In Vivo Imaging

In vivo imaging of luciferase activity was performed, as described previously [13] by injecting IFN-β^+/Δβ-luc^ mice intraperitoneally with D-luciferin (150 mg/kg). Mice were then monitored at 1-, 3-, 7- and 21-days post dose, using an IVIS 200 imaging system (IVIS Spectrum, Caliper Life Science, Waltham, WA, USA) (DaMBIC) and photon flux was quantified using Living Image 4.4 software (Caliper Life Science).

### 4.5. Tissue Processing

Mice were euthanized with an overdose of sodium pentobarbital (100 mg/kg, Glostrup Apotek, Glostrup, Denmark) and perfused with ice-cold PBS. For flow cytometry, CNS tissue was placed in ice-cold PBS. For histology, CNS tissue was post-fixed with 4% paraformaldehyde (PFA), immersed in 30% sucrose in PBS, then frozen and 16 μm thick tissue sections were cut on a cryostat (Leica, Copenhagen, Denmark). For reverse transcriptase-quantitative polymerase chain reaction (RT-qPCR), CNS tissues were placed in 0.5 mL TriZol Reagent (Ambion, Denmark) and stored at −80 °C until needed for RNA extraction [13].

### 4.6. Flow Cytometry

A single-cell suspension was obtained by forcing the CNS tissue through a 70 μm cell strainer (Falcon, Teterboro, NJ, USA) with Hank’s buffered salt solution (HBSS, Gibco, Waltham, MA, USA) supplemented with 2% fetal bovine serum (FBS, Merck, Darmstadt, Germany). Myelin was cleared by resuspending cells in 37% Percoll (GE Healthcare Bio-sciences AB, Uppsala, Sweden) in a buffer consisting of 45 mL 10x PBS, 3mL HCI, 132 mL water, pH 7.2, followed by centrifugation at 2500× *g* for 20 min at RT. The myelin layer was removed, and the cell pellet was washed. Cells were incubated in a blocking solution containing HBSS, 2% FBS, anti CD16/32 antibody (1 µg/mL, Clone 2.4G2, BD Biosciences, San Jose, CA, USA), and Syrian hamster IgG (50 μg/mL, Jackson Immuno Research Laboratories Inc., West Grove, PA, USA). The cells were then labeled with fluorophore-conjugated antibodies (BioLegend, Glostrup, Denmark): anti-CD45 (clone 30-F11), CD11b (M1/70), GR-1 (RB6–8C5), F4/80 (BM8), Ly6G (1A8) and Ly6C (HK1.4). Fluorescence was measured using an LSRII flow cytometer (BD Biosciences) with FACSDiva software (BD Biosciences) and analyzed with Flowlogic (Inivai Technologies, Pty, Mentone, Australia). 

### 4.7. Histology

To identify the cellular source of IFNβ, frozen brain sections from C57BL/6 and YFP/IFNβ reporter mice, intrathecally injected with AAV-GFP or AAV-CM, respectively, were incubated with the following primary antibodies: polyclonal rabbit anti-green fluorescent protein (GFP) (ab6556; Abcam, Cambridge, UK) to detect YFP and GFP, PE-conjugated rat anti-mouse CD45 (#103106, Biolegend, Denmark) or CY3-conjugated anti-glial fibrillary acidic protein (GFAP, C9205-2ML, Sigma, Denmark). Sections were then incubated with the appropriate secondary antibodies, including biotinylated goat anti rabbit IgG (H + L) (#64256, Abcam), followed by incubation with streptavidin–horseradish peroxidase (RPN1231V, GE Healthcare). GFP staining was developed using the TSA™ System (PerkinElmer, Skovlunde, Denmark) according to the manufacturer’s instructions. Nuclei were visualized by DAPI staining and the sections were mounted with gelvatol [5]. Isotype-matched control antibodies were used to verify the specificity of the primary antibodies (not shown).

Luxol Fast Blue (LFB) and cresyl violet staining were performed as described [31]. Images were acquired using an Olympus DP71 digital camera mounted on an Olympus BX51 microscope (Olympus, Tokyo, Japan). Images were combined using Adobe Photoshop CS3 (Adobe Systems Denmark A/S, Copenhagen, Denmark).

### 4.8. RNA Isolation and RT-qPCR

RNA extraction from brains and spinal cords was performed using TRIzol reagent in accordance with the manufacturer’s protocol. RNA was converted into cDNA using a high-capacity cDNA reverse transcription kit (Applied Biosystems, Foster City, CA, USA). RT-qPCR was performed using an ABI Prism 7300 sequence detection system (Applied Biosystems, Foster City, CA, USA) using the primers and probes described earlier. The following primer and probe sequences were used: IRF-7 (Forward CACCCCCATCTTCGACTTCA, Reverse CCAAAACCCAGGTAGATGGTGTA, Probe CACTTTCTTCCGAG AACT MGB), IFN-β (Forward GCGTTCCTGCTGTGCTTCTC, Reverse TTGAAGTCCGCCCTGTAGGT, Probe CGGAAATGTCAGGAGCT MGB), IFN-α (B+6+12+14) (Forward AGGATGTGACCTGCCTCAGACT, Reverse GCTGGGCAT-CCACCTTCTC, Probe CTCTCTCCTGCCTGAAG MGB), CCL2 (Forward TCTGGGCCT GCTGTTCACA, Reverse ACTCATT GGGATCATCTTGCT, Probe CTCAGCCAGATGCAG-TTMGB), CXCL10 (Forward GCCGTCATTTTCTGCCTCAT, Reverse GGCCCGTCAT-CGATATGG, Probe GGACTCAAGGGATCC MGB), IRF-9 (Forward ACAACTG-AGGCCACCATTAGAGA, Reverse CACCACTCG GCCACCATAG, Probe TGAACTC-AGACTACTCGCT MGB), IL-10 (Forward GGTTGCCAAGCCTTATCGGA, Reverse ACCTG-CTCCACTGCCTTGCT, Probe TGAGGCGCTGTCATCGATTTCTCCC TAMRA), IFN-γ (Forward CATTGAAAGCCTAGAAAGTCTGAATAAC, Reverse TGGCTCTGCAGGA-TTTTCATG, Probe TCACCATCCTTTTGC- CAGTTCCTCCAG MGB), STAT1 (Forward GGCCCAGTGGCTGGAAA, Reverse GTCGCAAACGAGACA-TCATAGG, Probe AAGA-CTGGGAGCACGCT MGB), IL-1β (Forward CTTGGGCCTCAAAGGAAAGAA, Reverse AAGACAAACCGTTTTTCCATCTTC, Probe AGCTGGAGAGTGTGGAT MGB), CXCL2 Mm00436450_m1 FAM. Ct values were determined, and the results are presented as genes of interest relative to the 18S rRNA (2^ΔCT^ method).

### 4.9. Statistical Analysis

The Rout test (Q = 1) was used to estimate significant outliers that were removed before further statistical testing. Data were tested for normal distribution and analyzed by a two-tailed non-parametric Student’s *t* test, followed by the Mann–Whitney test. All statistical analyses were performed using GraphPad Prism version 9 (Graphpad Software Inc., San Diego, CA, USA). The results are presented as means ± SEM. Values of *p* < 0.05 were considered significant.

## 5. Conclusions

We have demonstrated that sustained CNS-endogenous IFNβ production via intrathecal administration of AAV-CM promotes protection against EAE. Our results provide a basis for further studies aiming at elucidating the mechanism of sustained IFNβ signaling and protection in CNS inflammatory diseases.

## Data Availability

Not extra supporting data.

## Supplementary Materials

The following supporting information can be downloaded at: https://www.mdpi.com/article/10.3390/ijms231911292/s1.
