# Supplementary material for: Targeting Signaling Pathway Downstream of RIG-I/MAVS in the CNS Stimulates Production of Endogenous Type I IFN and Suppresses EAE
Source: Int J Mol Sci. 2022 Sep 25;23(19):11292. doi: 10.3390/ijms231911292 (PMC9570082; doi:10.3390/ijms231911292)
Supplement: Supplementary file 1 [file ijms-23-11292-s001.zip › ijms-1907903-supplementary.pdf]

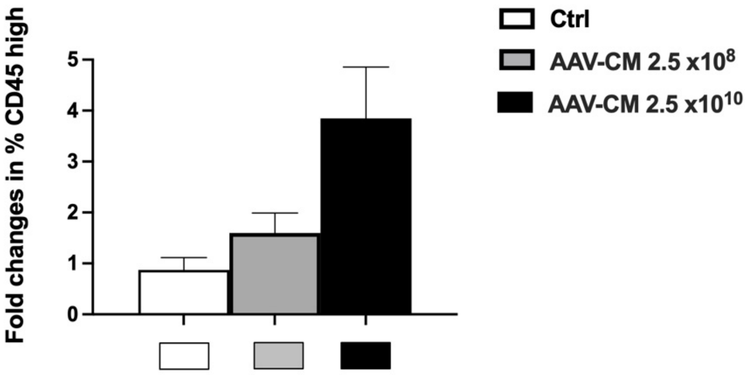

Figure S1. Intrathecal AAV-CM treatment induced CD45high infiltration in a dose-dependent manner.
